# In Situ Assembly of NiFe-LDH on Porous Sr-Doped LaCoO_3_ Scaffolds Using a Gel Template for High-Performance Oxygen Evolution Reaction

**DOI:** 10.3390/gels12050409

**Published:** 2026-05-08

**Authors:** Lina Zhang, Tian Fang, Changhai Liu, Wenchang Wang, Shiying Wang, Zhidong Chen

**Affiliations:** School of Materials Science and Engineering, Changzhou University, Changzhou 213164, China

**Keywords:** sol–gel method, Sr-doped LaCoO_3_, DFT, OER, heterostructure

## Abstract

This study reports a dual composition-interface engineering strategy for high-performance La_1−x_Sr_x_CoO_3_/NiFe-LDH hierarchical heterojunction. Porous La_1−x_Sr_x_CoO_3_ microspheres were synthesized through a gel route. Then it was used as an in situ–formed template to grow NiFe-LDH nanosheets. The hierarchical design inhibits nanosheet aggregation and ensures robust interfacial contact, mitigating the intrinsic instability of physical mixtures. The prepared composite displays superior OER performance in 1.0 M KOH, delivering an overpotential of 237.8 mV at 10 mA cm^−2^ and a Tafel slope of 85.06 mV dec^−1^. These values exceed those of the original samples and commercial RuO_2_ and the composite exhibits excellent long-term stability under harsh alkaline conditions. Complemented by DFT calculations, we further indicate that Sr doping coupled with the heterointerface induces substantial electronic structure reconstruction. This effectively switches the OER mechanism from conventional AEM to the thermodynamically more favorable LOM, overcoming the intrinsic scaling relation constraints of AEM.

## 1. Introduction

As global energy consumption increases and the dependence on fossil fuels is growing increasingly unsustainable, developing reliable electrochemical technologies for energy conversion and storage has become a top priority. Compared to the HER (Hydrogen Evolution Reaction), which involves only a two-electron process, the mechanism of OER (Oxygen Evolution Reaction) is significantly more complex. It entails a sequential four-electron-proton coupled transfer, proceeding through multiple elementary steps. These multi-step intermediate reactions greatly increase complexity, requiring higher external energy to overcome the kinetic barriers. This intrinsic characteristic severely limits the overall energy conversion efficiency of water electrolysis systems [1,2,3,4,5].

Perovskite oxides (ABO_3_) are promising, earth-abundant alternatives to noble metal electrocatalysts (e.g., IrO_2_, RuO_2_). Their tunable composition and flexible structure allow for A/B-site doping, which modulates electronic structures and oxygen vacancies to enhance activity [6,7,8,9,10]. Among perovskite oxides, lanthanum cobaltite (LaCoO_3_) stands out as an important candidate for the OER because of its intrinsically favorable electronic configuration, robust redox chemistry, and adjustable oxygen vacancy levels.

The lattice flexibility of both A and B sites offers a versatile platform for tailoring electronic states. For instance, partially substituting La^3+^ with Sr^2+^ at the A-site introduces hole doping, raising the average cobalt oxidation state from +3 to +4 and increasing the concentration of active Co^4+^ species. This electronic modulation not only alters surface adsorption energetics of intermediates but also enhances bulk electrical conductivity, thereby improving OER kinetics [6,7,8,10,11]. A-site engineering (e.g., with Ce, Sr, Ca, or Ba) in LaCoO_3_ is known to boost catalytic activity. Additionally, B-site substitution with transition metals such as Ni, Fe, or Mn directly affects the Co valence state, fine-tunes the oxygen adsorption free energy, and expands the pool of active sites, leading to superior catalytic efficiency [6,11]. The La_0.6_Sr_0.4_Co_0.8_Fe_0.2_O_3_ perovskite oxide achieves enhanced OER activity by leveraging A-site cation selective exsolution to modulate A-site cation defects [12]. For Ru-La_0.85_Sr_0.15_CoO_3_, the substitution of cobalt sites by ruthenium significantly enhances the OER catalytic activity due to the lattice incorporation effect [13]. Although doping modifies the perovskite electronic structure, low conductivity still limits charge transfer. To address this, integrating perovskites (e.g., LaCoO_3_) with layered double hydroxides (LDHs) or other supports (e.g., NiCo_2_O_4_, carbon, N-rGO and MoS_2_) has proven effective [14,15,16]. For example, the LaCoO_3_/NiCo_2_O_4_ heterostructure achieves 10 mA cm^−2^ at an overpotential of 353 mV and a Tafel slope of 59 mV dec^−1^ in alkaline media. This enhanced OER performance is attributed to increased electrochemically active surface area (ECSA), improved charge transfer, and interfacial synergy [17,18,19,20]. The LaCoO_3_@FeOOH core–shell heterostructure provides a larger specific surface area and more active sites, thereby enhancing the oxygen evolution reaction (OER) activity [21]. The LaCoO_3_/Co_3_O_4_ heterostructure leverages interface effects and vacancy defects to tune the electronic structure, significantly boosting OER activity [22].

In summary, although single-phase LaCoO_3_ suffers from low conductivity and limited active site density, its unique crystallographic features, compositional adaptability, and chemical stability make it a promising OER electrocatalyst. Two complementary strategies—doping and heterostructure construction—are commonly used to overcome these bottlenecks. Doping modifies the internal electronic environment via lattice defects, while heterostructuring tailors interfacial electronic structures. Their synergy significantly boosts catalytic performance [23,24,25]. For example, Sr doping in LaCoO_3_ induces oxygen vacancies, lattice distortion, and an upshifted O 2p band center, which strengthens Co 3d–O 2p hybridization and stabilizes a high-spin Co state. These electronic modifications simultaneously enhance OER activity and long-term stability by reinforcing Co–O bonding [26,27,28].

Transition metal-based layered double hydroxides (TM-LDHs), with NiFe-LDHs being the archetype, have emerged as the most promising electrocatalysts for the OER in alkaline environments [29]. The inherent adaptability of their brucite-like layers enables systematic engineering of both cationic ratios within the sheets and the nature of interlayer anions [30]. This structural plasticity is pivotal because it unveils a high density of edge-active sites while simultaneously promoting efficient ion diffusion and mass transport kinetics, culminating in a substantial enhancement of electrocatalytic efficiency [31]. Studies indicate that Co-doping triggers a beneficial reconstruction of the electronic structure at metal active sites in NiFe-LDHs. This modification significantly lowers the ΔG peak associated with the rate-determining step [32,33]. Thus, NiFe-LDHs rank among the most efficient OER catalysts in alkaline media. However, this exceptional activity often comes at the cost of structural stability; under high oxidative potentials, the characteristic layered architecture is prone to delamination or irreversible reconstruction, leading to performance degradation over time [34,35,36].

Literature reports indicate that La_1−x_Sr_x_CoO_3_ exhibits optimal OER activity within the range of x = 0.3–0.5, with x = 0.4 being the most widely reported composition. Therefore, we selected x = 0.4 as a representative doped sample for comparison with the undoped counterpart (x = 0) [37,38,39,40]. In this study, a perovskite-type lanthanum strontium cobalt oxide (LSC) with an abundant porous structure and high specific surface area was synthesized via a gel method. The LSC serves as a nanoscale scaffold that enables the in situ vertical growth of NiFe layered double hydroxide (NiFe-LDH) nanosheets through a hydrothermal process. The resulting open three-dimensional hierarchical composite architecture effectively suppresses the stacking of LDH nanosheets, exposing more accessible edge active sites. The synergistic electronic coupling between Co sites in LSC and Ni/Fe active centers in NiFe-LDH endows the composite with excellent electrocatalytic performance. This work proposes a “gel template induced in situ assembly” strategy to achieve, for the first time, the in situ growth of NiFe-LDH nanosheets on porous Sr-doped LaCoO_3_ microspheres, constructing a three-dimensional hierarchical heterostructure. Furthermore, combined with Density functional theory (DFT) calculations, it is revealed that the synergy between Sr doping and the heterointerface can switch the OER mechanism from the conventional AEM to LOM, thereby bypassing the limitation of adsorption energy scaling relations. This structure-electron dual-engineering strategy provides a new avenue for designing efficient and stable non-noble metal OER electrocatalysts.

## 2. Results and Discussions

### 2.1. Physicochemical Analysis

**Scheme 1:**
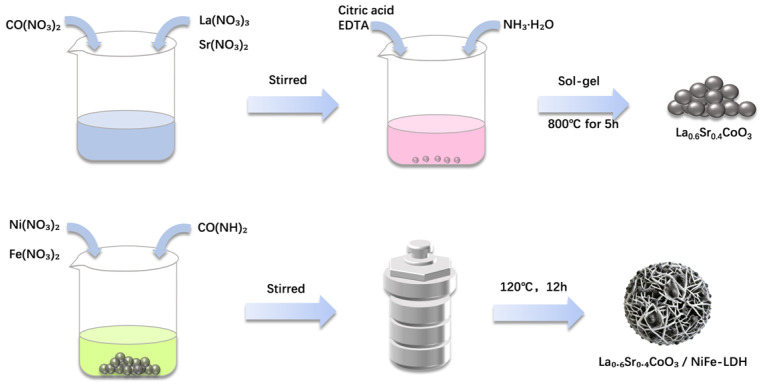
Illustration of the synthetic route for the La_0.6_Sr_0.4_CoO_3_/NiFe-LDH composite catalyst.

La_1−x_Sr_x_CoO_3_ samples were synthesized by a sol–gel strategy based on Sr(NO_3_)_2_, Co(NO_3_)_3_ and La(NO_3_)_3_ as metal precursors, with citric acid and EDTA. Then, NiFe-LDH nanosheets grew in situ on the La_1−x_Sr_x_CoO_3_ surface through a hydrothermal process. The synthetic procedure for the LSC/NiFe-LDH heterostructure is illustrated in Scheme 1.

**Figure 1:**
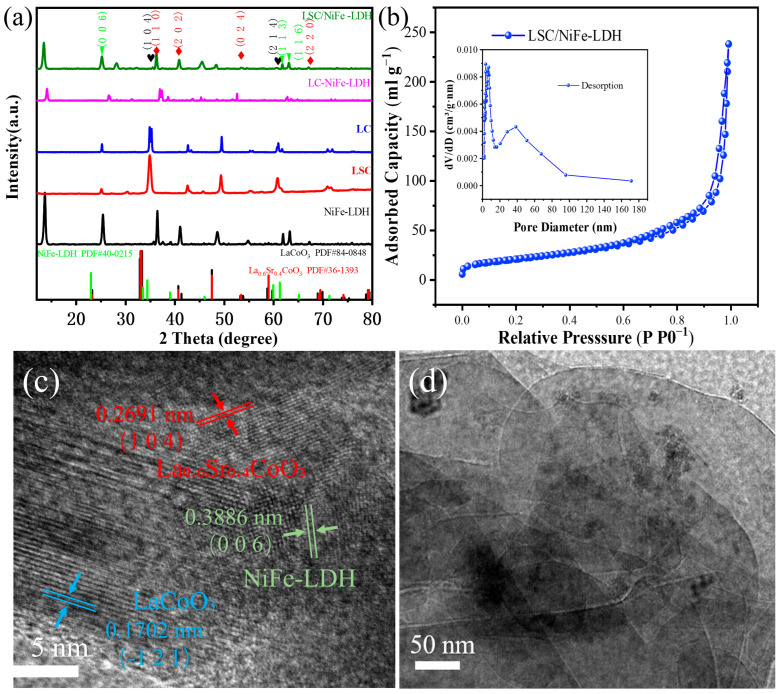
(**a**) XRD profiles of LC, LSC, NiFe-LDH, LC/NiFe-LDH, and LSC/NiFe-LDH. (**b**) Nitrogen adsorption–desorption isotherms and the corresponding BJH pore size distribution curves of LSC/NiFe-LDH. (**c**) HRTEM images of LSC/NiFe-LDH. (**d**) TEM images of LSC/NiFe-LDH.

To verify the effective doping of Sr into the LaCoO_3_ lattice and the formation of the LSC/NiFe-LDH composite. XRD (X-ray diffraction) was used to characterize the phase structures (Figure 1a). The diffraction patterns of the LC sample align well with the standard LaCoO_3_ phase (JCPDS No. 84-0848). Similarly, the Sr-doped LSC sample exhibits a perovskite structure corresponding to La_0.6_Sr_0.4_CoO_3_ (referenced against JCPDS No. 36-1393), to confirm the formation of the desired phases. Specifically, the primary diffraction peaks of LSC observed at 2θ = 32.98°, 33.178°, 47.46°, and 58.97° are indexed to the (012), (110), (202), and (024) crystal planes of the perovskite structure, respectively.

Notably, compared to the LC sample, the characteristic peaks of LSC exhibit a systematic shift toward lower 2θ angles. This negative shift indicates an expansion of the lattice parameters. Quantitative analysis of peak positions reveals a consistent shift of 0.2–0.6° toward lower angles for the perovskite diffraction peaks upon Sr doping (comparing LC with LSC), corresponding to an estimated lattice expansion of ~0.3–1.0%. While aliovalent Sr^2+^ substitution for La^3+^ can introduce oxygen vacancies and local structural distortions, the systematic and monotonic nature of the peak shifts across all crystallographic planes, combined with the absence of secondary phases or peak splitting, strongly supports that the dominant structural effect is isotropic lattice expansion due to the larger ionic radius of Sr^2+^ (~1.44 Å) compared to La^3+^ (~1.36 Å).

The XRD patterns of both LC/NiFe-LDH and LSC/NiFe-LDH composites show diffraction patterns that match well with both the perovskite substrate and the NiFe-LDH phase (JCPDS No. 40-0215). It verified the successful construction of the heterostructures. And the distinct peaks observed at 2θ = 22.97°, 61.26°, and 65.14° are indexed to the (006), (113), and (116) crystal planes of NiFe-LDH, respectively. It aligns well with the standard reference. Hydrothermal synthesis preserves the intrinsic crystalline structures of both components, as evidenced by the phase coexistence without any impurity peaks. Notably, a slight negative shift (toward lower 2θ angles) in the main diffraction peaks of the LSC/NiFe-LDH composite appears relative to the physical mixture of the individual components. This displacement is attributed to interfacial lattice strain and local structural reconstruction induced by strong electronic coupling at the heterojunction. Furthermore, the NiFe-LDH peaks in the hybrid architectures appear significantly broader and less intense compared to those of pristine NiFe-LDH nanosheets. According to the Scherrer equation, the observed peak broadening and intensity reduction suggest both a decrease in crystallite size and the introduction of microstrain. This is likely attributed to the porous perovskite scaffold inhibiting the growth and stacking of LDH nanosheets. Such structural features facilitate exposing more active edge sites and facilitating ion diffusion. Scherrer analysis based on the perovskite (110) diffraction peak yields crystallite sizes of 24–34 nm (Table S6), and the sharp diffraction peaks confirm the good crystallinity of all samples.

Transmission electron microscopy (TEM) and high-resolution transmission electron microscopy (HRTEM) further confirmed the successful construction of the LSC/NiFe-LDH heterostructure. The low-magnification TEM image (Figure 1d) reveals a typical ultrathin, wrinkled sheet-like morphology, consistent with the nanosizing/exfoliation of NiFe-LDH during compositing. This corroborates the XRD results (weakened intensity and peak broadening), indicating a reduced degree of ordered layer stacking. In the HRTEM image (Figure 1c), three distinct sets of lattice fringes are clearly observed: the fringe spacing of 0.2691 nm is assigned to the (104) plane of La_0.6_Sr_0.4_CoO_3_ (LSC); 0.3886 nm corresponds to the (006) plane of NiFe-LDH, matching its layered features; and 0.1702 nm is attributed to the (−121) plane of LaCoO_3_. The close contact and high crystallinity of these phases not only demonstrate the successful integration of the LSC perovskite and NiFe-LDH but also align with the XRD findings of phase coexistence. Furthermore, the well-defined heterointerface suggests strong interfacial coupling, which echoes the slight peak shifts induced by lattice strain in the XRD patterns, providing direct structural evidence for the synergistic electrocatalytic effect.

The N_2_ adsorption–desorption isotherms of LSC-NiFe-LDH exhibit a typical type-IV profile with a distinct hysteresis loop, indicating a rich mesoporous structure. The corresponding pore size distribution curve (Figure 1b) shows a primary pore size range of 2–20 nm with an average diameter of 7.65 nm, further confirming its mesoporous nature. The material possesses a BET specific surface area of 74.62 m^2^/g. Notably, the t-plot analysis reveals a negligible micropore volume, and the t-plot surface area is nearly identical to the BET value, suggesting that the surface area is predominantly contributed by mesopores. Such a mesoporous architecture not only provides abundant exposed active sites for the OER but also facilitates electrolyte infiltration and oxygen bubble release, thereby enhancing mass transport efficiency and boosting the overall OER catalytic performance.

**Figure 2:**
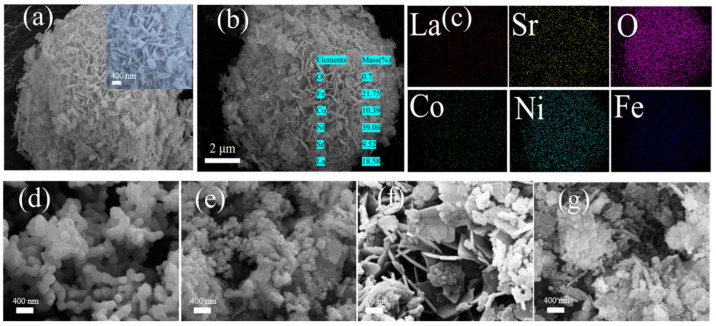
SEM image of (**a**) LSC/NiFe-LDH; (**b**,**c**) EDS mapping images of LSC/NiFe-LDH, showing the elemental distribution of La, Sr, Co, O, Ni, and Fe; SEM micrographs of (**d**) LC, (**e**) LSC, (**f**) NiFe-LDH, and (**g**) LC/NiFe-LDH.

To further investigate the morphology and elemental distribution of the composites, we used SEM (Scanning Electron Microscope) coupled with EDS mapping on the LSC/NiFe-LDH sample. As depicted in Figure 2c, La, Sr, Co, O, Ni, and Fe elements are homogeneously distributed across the entire surface, which confirms the uniform integration of the constituent phases. For establishing stable interfacial contacts and fostering synergistic cooperative effects, compositional homogeneity is crucial. Energy-dispersive X-ray spectroscopy (EDS) analysis (Figure S9, Table S5) gives a Sr/(La + Sr) atomic ratio of 0.34 for the LSC/NiFe-LDH composite. Given the semi-quantitative nature of EDS and the fact that the LDH coating may partially screen the signal from the perovskite substrate, this value is reasonably close to the nominal composition of x = 0.4. We therefore conclude that Sr has been successfully incorporated into the perovskite lattice.

The SEM images (Figure 2d–g) reveal distinct morphological features that pristine LC and LSC consist of aggregated particles, but pure NiFe-LDH displays a characteristic sheet-like layered structure. These features coexist in the hybrid composites (LC/NiFe-LDH and LSC/NiFe-LDH), verifying the successful combination of the two components. The composites exhibit a unique hierarchical architecture. Unlike the bare LSC substrate, the composite surface is densely covered with vertically aligned NiFe-LDH nanosheets to construct a porous three-dimensional network. Although the LSC core is partially shielded by the dense LDH overlayer, this nanosheet-assembled structure offers distinct advantages for electrocatalysis. It significantly expands the specific surface area and maximizes the exposure of active edge sites. Furthermore, the interconnected porous channels facilitate rapid electrolyte diffusion and efficient oxygen bubble release. The intimate contact between the conductive LSC substrate and the LDH nanosheets signifies the formation of a robust heterojunction, which is pivotal for accelerating charge transfer kinetics.

In comparison, the LSC/NiFe-LDH (Figure 2b) sample exhibits a denser stacking of nanosheets than the LC/NiFe-LDH (Figure 2a) counterpart. It indicates a higher loading and more uniform growth of NiFe-LDH on the Sr-doped scaffold. This enhanced coverage is expected to enlarge the interfacial contact area and expose a greater number of electroactive sites, thus substantially boosting the overall catalytic performance.

**Figure 3:**
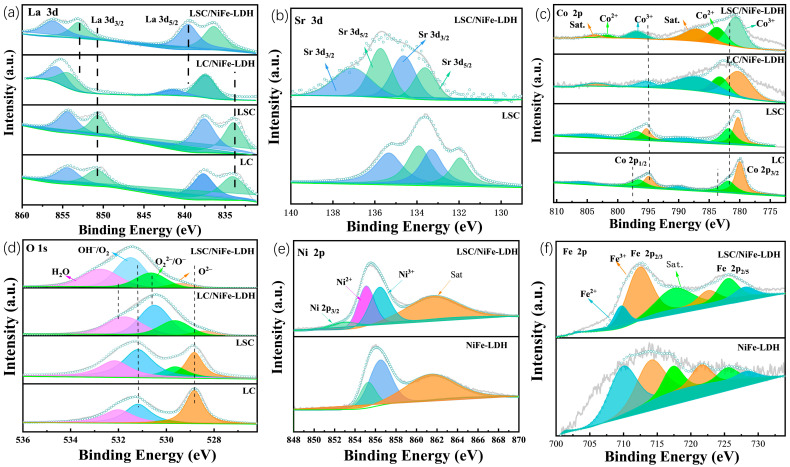
XPS Characterization: (**a**) La 3d XPS spectra of LC, NiFe-LDH, LC/NiFe-LDH, and LSC/NiFe-LDH. (**b**) Sr 3d XPS spectra of NiFe-LDH and LSC/NiFe-LDH. (**c**) Co 2p XPS spectra of LC, NiFe-LDH, LC/NiFe-LDH, and LSC/NiFe-LDH. (**d**) O 1s XPS spectra of LC, NiFe-LDH, LC/NiFe-LDH, and LSC/NiFe-LDH. (**e**) Fe 2p XPS spectra of NiFe-LDH and LSC/NiFe-LDH. (**f**) Ni 2p XPS spectra of NiFe-LDH and LSC/NiFe-LDH.

The surface composition and elemental valence states of the catalysts were characterized by XPS (X-ray photoelectron spectroscopy). As shown in Figure 3a, the La 3d spectrum of the pristine LC sample exhibits two characteristic spin–orbit doublets, La 3d_5/2_ and 3d_3/2_. Two main peaks are centered at binding energies of 833.79 eV and 850.81 eV, respectively, accompanied by distinct satellite peaks arising from charge-transfer transitions between O 2p and La 4f orbitals. When Sr is doped into the sample (forming the LSC composition), a slight shift in the La 3d binding energies is detected. Compared to their respective precursors, both the LC/NiFe-LDH and LSC/NiFe-LDH composites show a positive shift of about 2.0 eV in the La 3d_5/2_ and 3d_3/2_ peaks. This positive binding energy shift indicates a depletion of electron density around the La sites, suggesting electron transfer from La to the adjacent NiFe-LDH phase or oxygen species. This finding provides compelling evidence for the modulation of the electronic structure at the heterointerface. The high-resolution Sr 3d spectrum (Figure 3b) was fitted with a four-component peak. For the LSC sample, two peaks are observed at 131.83 eV and 133.31 eV. These correspond to states within the perovskite lattice. Two additional signals are detected at higher binding energies (133.63 eV and 134.72 eV). These likely arise from surface Sr species or those in distinct coordination environments induced by doping. Upon coupling LSC with NiFe-LDH, the Sr 3d peaks also display a positive shift of 1.9 eV, in agreement with the La 3d results. This consistent shift supports strong electronic coupling between interactions and charge redistribution across the composite interface.

Furthermore, Figure 3c presents the high-resolution Co 2p spectrum, which comprises two main spin–orbit components (Co 2p_3/2_ and Co 2p_1/2_) along with their corresponding satellite features. Notably, both the Co 2p_3/2_ and Co 2p_1/2_ envelopes are fitted with two distinct sub-peaks, suggesting mixed cobalt valence states (e.g., Co^3+^ and Co^4+^) within the lattice. In the LC sample, the Co 2p spectrum is deconvoluted into two sets of spin–orbit doublets corresponding to Co^3+^ (779.88/794.88 eV) and Co^2+^ (781.79/796.49 eV) species. The LSC sample exhibits subtle variations in these spectral features. Notably, upon coupling both LC and LSC with NiFe-LDH, the Co 2p_3/2_ and Co 2p_1/2_ peaks undergo a positive binding energy shift. This shift suggests that the introduction of NiFe species modulates the oxidation state of Co within the perovskite lattice. Quantitative assessment of the peak areas reveals that the Co^3+^/(Co^2+^+Co^3+^) ratios for LC, LSC, LC/NiFe-LDH, and LSC/NiFe-LDH are 0.503, 0.527, 0.591 and 0.627, respectively. These results indicate a predominance of Co^3+^ species and an elevated average oxidation state of Co in the LSC/NiFe-LDH composite. Such electronic modulation is attributed to the interfacial charge polarization effect at the heterostructure interface, which enhances cationic redox activity and synergistically boosts electrocatalytic performance.

The O 1s spectrum (Figure 3d) can be fitted into four components: lattice oxygen (O^2−^), high-valence oxygen species (associated with O_2_^2−^/O^−^), surface-adsorbed hydroxyl/oxygen groups (-OH/O_2_), and adsorbed water (H_2_O). Upon successful material synthesis, the O 1s peaks in both LC and LSC shift toward higher binding energy. This confirms the formation of a strong heterogeneous interface, which enhances the stability of the metal-oxygen covalent bond. Given the critical role of oxygen sites in catalysis, the presence of high-valence oxygen species is closely linked to the generation of surface oxygen vacancies [41]. As shown in Table S2, the relative proportion of lattice oxygen decreases progressively across the LC, LSC, LC/NiFe-LDH, and LSC/NiFe-LDH series, while the content of high-valence oxygen species increases correspondingly from 5.1% to 14.9%, 27.1%, and finally 30.1%. This trend implies the active participation of lattice oxygen in the OER, aligning well with the Lattice Oxygen Mechanism (LOM). Thus, A-site doping combined with heterostructure construction effectively regulates oxygen vacancy concentrations, thereby optimizing electrocatalytic activity.

High-resolution Ni 2p and Fe 2p spectra are presented in Figure 3e,f. The Fe 2p spectrum displays characteristic peaks at 709.8 eV and 712.8 eV, assigned to Fe^2+^ and Fe^3+^ species in the NiFe-LDH phase, respectively. Similarly, the Ni 2p spectrum exhibits distinct peaks at 855.0 eV and 856.5 eV, corresponding to Ni^2+^ and Ni^3+^ oxidation states. Upon integration with the perovskite substrate, both Ni 2p and Fe 2p peaks shift to higher binding energies, indicating electron density depletion (partial oxidation) of the Ni and Fe species within the heterostructure. These observations demonstrate strong electronic coupling between the NiFe-LDH nanosheets and the LSC substrate. Such interfacial charge polarization facilitates the adsorption of reaction intermediates, increases the density of active sites, and significantly enhances the overall catalytic activity.

### 2.2. Electrochemical Analysis

**Figure 4:**
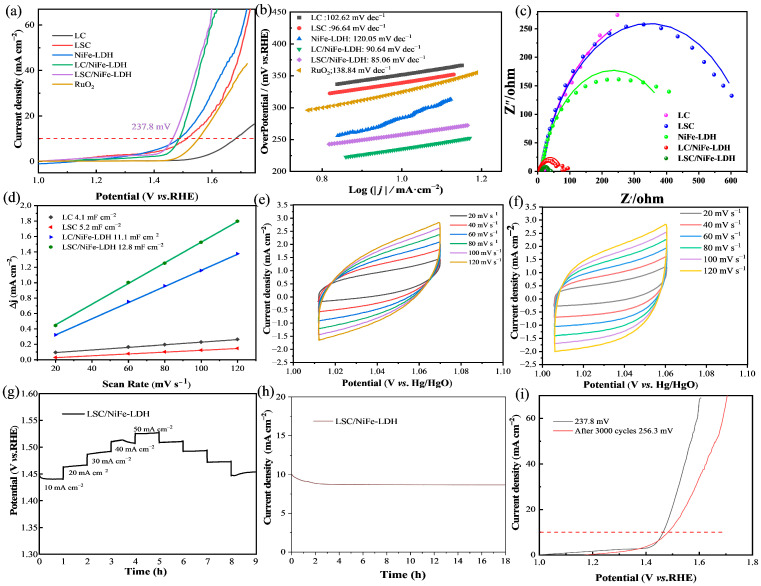
(**a**) LSV curves at 5 mV s^−1^ in 1.0 M KOH; (**b**) Tafel plots; (**c**) Nyquist plots at 1.55 V vs. RHE; (**d**) C_dl_ values derived from CV scans at 20–120 mV s^−1^; (**e**,**f**) CV curves at different scan rates for LC/NiFe-LDH and LSC/NiFe-LDH; (**g**–**i**) stability tests of LSC/NiFe-LDH by different methods.

The OER activity of the catalysts was assessed in a three-electrode system with 1 M KOH as the electrolyte. As shown in the LSV (linear sweep voltammetry) profiles (Figure 4a), the LSC/NiFe-LDH composite exhibits superior activity, requiring an overpotential of only 237.8 mV to deliver a current density of 10 mA cm^−2^. This value is markedly lower than those of the pristine counterparts (LC: 455.8 mV; LSC: 285.9 mV; NiFe-LDH: 259.9 mV), the LC/NiFe-LDH hybrid (260.1 mV), and even the commercial RuO_2_ benchmark (330.2 mV). Such a significant reduction in overpotential underscores the efficacy of constructing the LSC/NiFe-LDH heterostructure in boosting OER activity.

Kinetic analysis further corroborates these findings. The Tafel slope calculated for LSC/NiFe-LDH is 85.06 mV dec^−1^ (Figure 4b), which is smaller than those of LC (102.62 mV dec^−1^), LSC (96.64 mV dec^−1^), NiFe-LDH (120.05 mV dec^−1^), LC/NiFe-LDH (90.64 mV dec^−1^) and RuO_2_ (138.84 mV dec^−1^). This small Tafel slope suggests faster reaction kinetics and a lower kinetic barrier for the LSC/NiFe-LDH catalyst. To determine the ECSA, cyclic voltammetry (CV) tests were performed at scan rates between 20 and 120 mV s^−1^ (Figure 4e,f). The double-layer capacitance (C_dl_), determined from the slope of the current density–scan rate curve, serves as a proxy for ECSA. As shown in Figure 4d, LSC/NiFe-LDH yields a C_dl_ of 12.8 mF cm^−2^, substantially exceeding those of LC (4.1 mF cm^−2^), LSC (5.2 mF cm^−2^), and LC/NiFe-LDH (11.1 mF cm^−2^). This enhanced C_dl_ indicates a larger ECSA and a greater abundance of exposed active sites, directly contributing to the improved catalytic performance. It should be noted that all composite samples were synthesized with identical precursor loadings (0.6 mmol perovskite, 1.8 mmol Ni^2+^, 0.9 mmol Fe^3+^), ensuring comparable theoretical NiFe-LDH loadings. SEM observations (Figure 2a) further reveal similar surface coverage by LDH nanosheets across different composites. Moreover, the C_dl_ of LSC/NiFe-LDH (12.8 mF cm^−2^) is only ~15% higher than that of LC/NiFe-LDH (11.1 mF cm^−2^), while the overpotential decreases by 22 mV and the Tafel slope by 5.6 mV dec^−1^. This disproportionate enhancement suggests that the improved OER performance originates primarily from enhanced intrinsic activity due to Sr doping and interfacial electronic coupling, rather than mere differences in LDH loading.

Electrochemical impedance spectroscopy (EIS) was applied to explore the charge transfer dynamics (Figure 4c). The Nyquist plots show that LSC/NiFe-LDH exhibits the smallest semicircle diameter, representing the lowest charge-transfer resistance (R_ct_) among all tested samples. This diminished R_ct_ confirms facilitated interfacial charge transport, aligning perfectly with its outstanding OER activity.

Long-term stability was assessed via multi-step chronopotentiometry (Figure 4g), where the potential response remained stable under stepwise current densities from 10 to 50 mA cm^−2^ (1 h per step). After 9 h of operation, returning the current to 10 mA cm^−2^ resulted in negligible potential fluctuation, demonstrating excellent robustness against current shocks. Furthermore, chronopotentiometry tests (Figure 4h) at a constant 10 mA cm^−2^ for 18 h showed virtually no potential decay. Post-stability LSV curves recorded after 3000 CV cycles (Figure 4i) remained nearly identical to the initial profiles. Collectively, these results confirm the exceptional structural durability and long-term operational stability of the LSC/NiFe-LDH catalyst in alkaline media. To uncover the origin of the excellent stability of LSC/NiFe-LDH, we characterized the samples before and after the reaction in 1 M KOH using XRD and TEM (Supplementary Materials Figures S12 and S13). The XRD patterns before and after the reaction are nearly identical (Figure S12), indicating no significant change in crystal structure. Similarly, TEM images (Figure S13a–b) show that the catalyst morphology remains almost unchanged. These results demonstrate that the physical and chemical properties of the catalyst are well preserved after the reaction. Thus, LSC/NiFe-LDH exhibits outstanding catalytic stability and holds great promise for practical applications.

To evaluate the materials’ water-splitting performance, the prepared samples were loaded onto a nickel foam substrate and used as the anode and cathode of the electrolyzer, respectively; in this study, an alkaline water electrolyzer was constructed, with 1 M potassium hydroxide (KOH) solution selected as the electrolyte. Under a current density of 10 mA cm^−2^, the operating voltage of the LSC/NiFe-LDH||LSC/NiFe-LDH total water splitting system was 1.77 V, demonstrating its catalytic performance for total water splitting. Furthermore, as shown by the chronopotentiometric test results (Supplementary Materials Figure S11), the LSC/NiFe-LDH||LSC/NiFe-LDH electrode system exhibited catalytic durability of up to 15 h in the chronopotentiometric test.

Compared with previously reported analogous catalysts (Table S4), the LSC/NiFe-LDH heterostructure synthesized in this work exhibits a lower overpotential of only 237.8 mV at 10 mA cm^−2^, demonstrating superior OER catalytic performance. This significant enhancement can be attributed to the intrinsic structural advantages of the material, including Sr doping that optimizes the electronic structure of LaCoO_3_ (as confirmed by XPS and DFT calculations), the porous architecture derived from the gel template method that increases the electrochemically active surface area, and the interfacial electronic coupling that facilitates charge transfer and activates the lattice oxygen mechanism (LOM), thereby substantially boosting the intrinsic catalytic activity.

### 2.3. DFT Calculations

**Figure 5:**
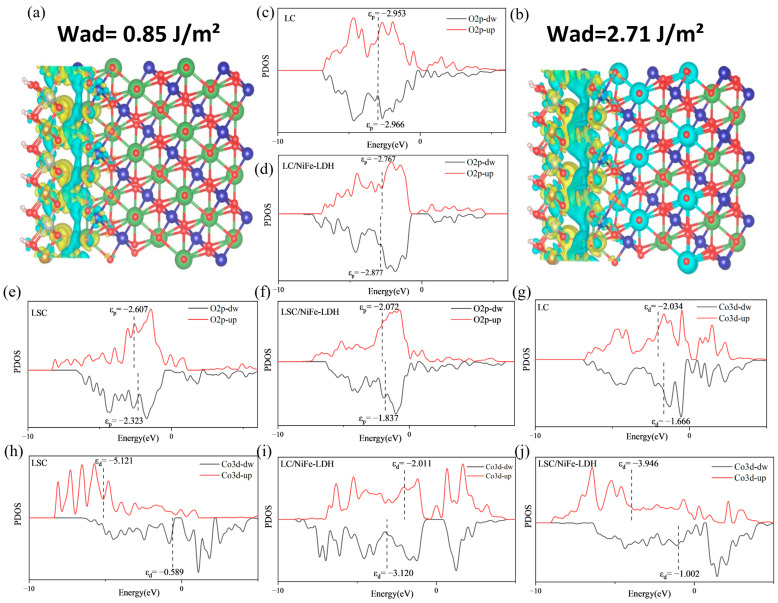
DFT results: Interfacial interaction and electronic structure analysis. Charge density difference plots of (**a**) LC/NiFe-LDH and (**b**) LSC/NiFe-LDH heterostructures (yellow: charge accumulation, cyan: charge depletion); (**c**–**f**) Spin-resolved PDOS of O 2p orbitals and the *Ɛ_p_* for LC, LSC, LC/NiFe-LDH, and LSC/NiFe-LDH; (**g**–**j**) Spin-resolved PDOS of Co 3d orbitals and the corresponding d- band center *Ɛ_d_* for the corresponding samples.

DFT calculations using the Vienna Ab initio Simulation Package (VASP, version number-VASP 6.3, Vienna, Austria) (details in Supplementary Materials) were performed to clarify the atomic-scale synergy between Sr doping and heterostructure construction for OER. Interface strength was assessed by adhesion work (W_ad_) and charge density difference. As shown in Figure 5a,b, the LSC/NiFe-LDH interface exhibits much denser charge accumulation/depletion than LC/NiFe-LDH, with electron transfer from metal sites to interfacial oxygen, indicating strengthened electronic coupling. Accordingly, W_ad_ of LSC/NiFe-LDH reaches 2.71 J m^−2^, far exceeding that of LC/NiFe-LDH (0.85 J m^−2^), confirming robust interface binding that underpins the excellent durability (Figure 4g–i).

This strong coupling induces electronic reconstruction, evidenced by oxygen vacancy formation energy (E_ov_, Table S3) and Projected Density of States (PDOS). LSC/NiFe-LDH shows the lowest E_ov_ (1.04 eV), much lower than LC (1.89 eV), LSC (1.37 eV), and LC/NiFe-LDH (1.73 eV), indicating facile lattice oxygen activation. As shown in Figure 5c–j, both Sr doping and NiFe-LDH coupling upshift the O 2p band center toward the Fermi level. From LC to LSC/NiFe-LDH, the spin-down and spin-up O 2p band centers cumulatively shift upward by >1.1 eV, aligning with XPS results where high-valence oxygen species increase from 5.1% to 30.1% (Table S2). Spin-resolved PDOS of Co 3d orbitals (Figure 5) reveals that Sr doping induces significant spin polarization (Ɛ_d↑_ shifts to −5.121 eV, Ɛ_d↓_ to −0.589 eV), reflecting a higher Co oxidation state. After heterojunction formation, Co–O–Ni/Fe bridging triggers orbital hybridization, shifting Ɛ_d↓_ to −1.002 eV and Ɛ_d↑_ to −3.946 eV, mitigating spin asymmetry. This “high-valence + spin-orbital reconstruction” weakens oxygen intermediate adsorption and promotes lattice oxygen activation, establishing a favorable basis for the lattice oxygen mechanism (LOM). Interfacial coupling is also evident on the NiFe-LDH side, where increased binding energies of Ni 2p and Fe 2p (Figure 3e–f) agree well with the d-band center evolution from DFT (Figures S1–S6).

**Figure 6:**
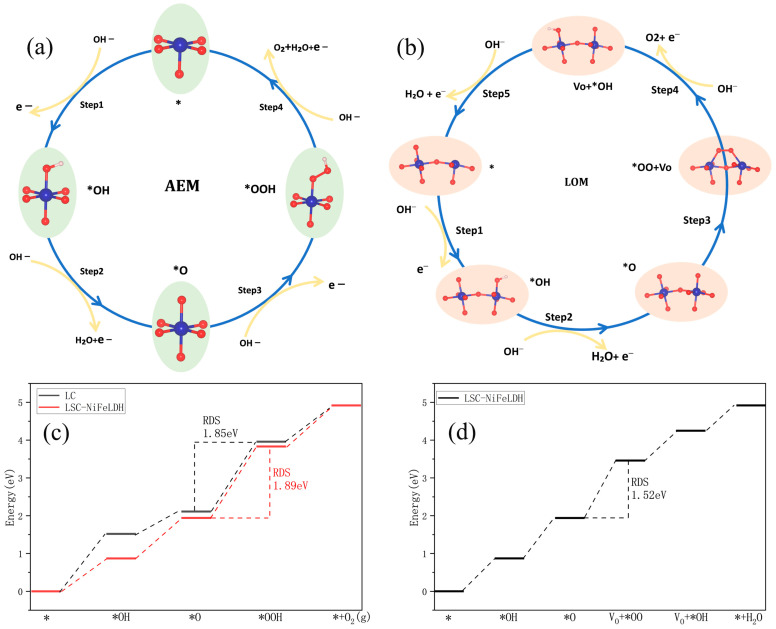
Atomic-scale insight into the OER mechanism: (**a**) Schematic of the AEM pathway at Co sites in LC; (**b**) Schematic of the LOM pathway at Co sites in LSC/NiFe-LDH; (**c**) Free energy diagram of LC along the AEM pathway; (**d**) Comparison of free energy diagrams for LSC/NiFe-LDH following both AEM and LOM pathways (computational models are shown in Figures S7 and S8). Note: The asterisks (*) on the x-axis in Figure 6c,d represent the catalysts in the reaction process.

To confirm the mechanistic transition, we evaluated both the adsorbate evolution mechanism (AEM) and the LOM pathways (Figure 6a,b; computational models in Figures S7 and S8). For pristine LC following AEM, the rate-determining step (*O → *OOH) has a barrier of 1.85 eV (Figure 6c). For LSC/NiFe-LDH, the AEM barrier increases to 1.89 eV due to overly strong adsorption, whereas the LOM pathway drastically reduces the barrier to 1.52 eV (Figure 6d), corresponding to a theoretical overpotential of 0.29 V, in excellent agreement with the experimental value (237.8 mV). Thus, Sr doping and heterointerface engineering synergistically activate the thermodynamically favorable LOM, overcoming the scaling limits of traditional AEM.

## 3. Conclusions

In this study, porous perovskite-type lanthanum strontium cobalt oxide (LSC) was synthesized via a sol–gel method and used as a nanoscale scaffold to achieve in situ vertical growth of NiFe layered double hydroxide (NiFe-LDH) nanosheets through a hydrothermal process, forming an open three-dimensional hierarchical composite structure. Benefiting from the “gel template induced in situ assembly” strategy, NiFe-LDH nanosheets are successfully grown in situ on porous Sr-doped LaCoO_3_ microspheres for the first time, forming a three-dimensional hierarchical heterostructure. Combined with DFT calculations, it is revealed that the synergy between Sr doping and the heterointerface switches the OER mechanism from the conventional AEM to LOM, thereby bypassing the limitation of adsorption energy scaling relations. Consequently, electrochemical measurements confirm the superior OER performance of the as-constructed heterojunction. In 1.0 M KOH, the catalyst achieves a current density of 10 mA cm^−2^ at a low overpotential of only 237.8 mV with a small Tafel slope of 85.06 mV dec^−1^, significantly outperforming the individual components and commercial RuO_2_. Moreover, the catalyst demonstrates remarkable long-term operational stability under aggressive alkaline conditions. This structure-electron dual-engineering strategy provides a new approach for designing non-noble metal OER electrocatalysts.

## 4. Materials and Methods

### 4.1. Experiment Part

#### 4.1.1. Sol–Gel Synthesis of Perovskite-Type La_1−x_Sr_x_CoO_3_ Oxides

In a typical synthesis, La_1−x_Sr_x_CoO_3_ samples (LC: x = 0; LSC: x = 0.4) were prepared by dissolving Co(NO_3_)_2_·6H_2_O (10 mmol), La(NO_3_)_3_·6H_2_O (10 mmol for LC; 6 mmol for LSC), and Sr(NO_3_)_2_ (0 mmol for LC; 4 mmol for LSC) in 100 mL deionized water. Citric acid (24 mmol) and EDTA (16 mmol) were added, followed by pH adjustment to 8–9 with aqueous ammonia. The EDTA/citric acid sol–gel method was employed to ensure a homogeneous distribution of cations. The mixture was stirred at 90 °C to form a gel, which was dried at 250 °C for 5 h. The resulting precursor was ground and calcined in air at 800 °C for 5 h (ramping rate: 5 °C min^−1^) to obtain the final perovskite powders [42].

#### 4.1.2. In Situ Growth of La_1−x_Sr_x_CoO_3_/NiFe-LDH Composites

In a typical synthesis, 1.8 mmol Ni(NO_3_)_2_ ·6H_2_O, 0.06 mmol urea (CO(NH_2_)_2_), 0.9 mmol Fe(NO_3_)_3_ ·9H_2_O and 0.6 mmol LC (or LSC) powder were dispersed in 40 mL deionized water. After stirring for 0.5 h, the mixture was sealed in an autoclave and heated at 120 °C for 12 h. Upon natural cooling, the precipitate was collected via centrifugation, washed thoroughly with deionized water, and dried at 70 °C for 12 h to obtain the target composites.

### 4.2. Physicochemical Characterization of Materials

Crystal structures were identified by X-ray diffraction (XRD, Rigaku D/MAX-2500, Rigaku Corporation, Tokyo, Japan) with Cu Kα, (λ = 1.54056 Å) in the 2θ range of 5–80°. Surface chemical states and elemental composition were investigated via X-ray photoelectron spectroscopy (XPS, Thermo Fisher ESCALAB 250Xi, Thermo Fisher Scientific, Waltham, MA, USA), with binding energies calibrated to the C 1s peak (284.8 eV). Morphological features were observed using both a JEOL JSM-6360LA Scanning Electron Microscope (SEM, JEOL JSM-6360LA, JEOL Ltd., Tokyo, Japan) and a Carl Zeiss SUPRA 55 Field Emission Scanning Electron Microscope (FE-SEM, Carl Zeiss SUPRA 55, Carl Zeiss AG, Oberkochen, Germany). Transmission electron microscopy (TEM, JEM-2100F, JEOL Ltd., Tokyo, Japan) and high-resolution transmission electron microscopy (HR-TEM, same instrument) were performed to identify the phase, crystal structure, and grain size of the materials. Density functional theory (DFT) calculations on a heterojunction interface provided the projected density of states (DOS), charge density difference, and adsorption energies, offering electronic-level insights into the improved performance.

### 4.3. Electrochemical Characterization of Materials

Electrochemical tests were carried out on a CHI760E workstation (CH Instruments, Shanghai, China), except for electrochemical impedance spectroscopy (EIS) measurements, which were recorded on a Parstat 3000 (Princeton Applied Research, Oak Ridge, TN, USA). For electrode preparation, 1.5 mg of catalyst and 1.5 mg of XC-72 conductive carbon were dispersed in 190 μL H_2_O and 60 μL Ethanol, followed by the addition of 10 μL Nafion (0.5 wt%). After ultrasonication for 80 min, 4 μL of the ink was drop-cast onto a 3 mm GCE and dried. The resulting catalyst loading was 0.326 mg cm^−2^ [42].

Electrochemical OER measurements were performed in 1.0 M KOH using a three-electrode system: a catalyst-loaded glassy carbon working electrode, a Hg/HgO reference electrode, and a graphite rod counter electrode. Potentials were calibrated to the RHE scale using the equation:

E_RHE_ = E_Hg/HgO_ + 0.059 pH + 0.098

where E_Hg/HgO_ is the measured potential and pH is that of the electrolyte [42].

OER polarization curves were obtained via linear sweep voltammetry. Tafel slopes (b) were derived from the linear fit of η = b log j + a. C_dl_ values were extracted from cyclic voltammetry scans at rates of 20–120 mV s^−1^ (step: 20 mV s^−1^). Assuming a specific capacitance (C_s_) of 40 μF cm^−2^, the ECSA was calculated as C_dl/_C_s_. EIS spectra were recorded from 100 kHz to 0.1 Hz (amplitude: 5 mV). Excitation potential is 1.55 V vs. RHE (Reversible Hydrogen Electrode). The EIS measurement was performed at 1.55 V vs. RHE, a potential at the foot of the OER polarization curve where no significant oxygen bubble evolution occurs. This allows for reliable determination of charge-transfer resistance (R_ct_) without interference from gas evolution.

Stability was assessed by: (1) an accelerated degradation test (3000 CV cycles at 100 mV s^−1^) monitoring Δη; (2) 18 h chronopotentiometry at 10 mA cm^−2^; (3) multi-step chronopotentiometry.

## Data Availability

Data will be available upon reasonable request to the corresponding author.

## Supplementary Materials

The following supporting information can be downloaded at: https://www.mdpi.com/article/10.3390/gels12050409/s1, Figure S1. Spin-resolved PDOS of Ni 3d orbitals and the corresponding d-band center (εd) for pristine NiFe-LDH (Figure S2. LC/NiFe-LDH, Figure S3. LSC/NiFe-LDH); Figure S4. Spin-resolved PDOS of Fe 3d orbitals and the corresponding d-band center (εd) for pristine NiFe-LDH (Figure S5. LC/NiFe-LDH, Figure S6. LSC/NiFe-LDH); Figure S7. Structures of the key intermediates on the associative electrode mechanism (AEM) pathway of LC; Figure S8. Structures of the key intermediates on the associative electrode mechanism (AEM) and lattice oxygen mechanism (LOM) pathway of LSC/NiFe-LDH; Figure S9. EDS Quantitative Analysis Spectrum of LSC/NiFe-LDH Composite Material; Figure S10 XPS Characterization: Wide-scan XPS survey of the LSC/NiFe-LDH sample; Figure S11 Chronopotentiometry curves of the as-prepared electrocatalysts at a constant current density of 10 mA cm^−2^; Inset: Overall water splitting polarization curves; Figure S12. XRD comparison of LSC/ NiFe-LDH before and after reaction; Figure S13. TEM comparison of LSC/NiFe-LDH before (a) and after (b) reaction; Table S1. Calculated oxygen vacancy formation energies (Eov); Table S2. Relative abundance of various oxygen species based on O 1s XPS analysis; Table S3. Comparison of ECSA values for different OER catalysts in alkaline solutions; Table S4. OER activities of this work at 10 mA cm^−2^ was compared with that of previously reported similar catalysts in 1 M KOH solution; Table S5. EDS quantitative analysis of the LSC/NiFe-LDH composite based on mapping data in Figure S9; Table S6. Crystallite sizes calculated from the perovskite (110) diffraction peak using the Scherrer equation.
