# Supplementary material for: In Situ Assembly of NiFe-LDH on Porous Sr-Doped LaCoO3 Scaffolds Using a Gel Template for High-Performance Oxygen Evolution Reaction
Source: Gels. 2026 May 8;12(5):409. doi: 10.3390/gels12050409 (PMC13205260; doi:10.3390/gels12050409)
Supplement: Supplementary file 1 [file gels-12-00409-s001.zip › gels-4237081-supplementary.pdf]

In Situ Assembly of NiFe-LDH on Porous Sr-Doped $LaCoO_3$ Scaffolds Using a Gel Template for High-Performance Oxygen Evolution Reaction

Lina Zhang, Tian Fang, Changhai Liu, Wenchang Wang, Shiying Wang, Zhidong Chen[1, *]
School of Materials Science and Engineering, Changzhou University;
* Correspondence: zdchen@cczu.edu.cn

Computation methods

DFT calculations were performed by using the Vienna Ab-initio Simulation Package (VASP). The exchange–correlation interactions were described by generalized gradient approximation (GGA) with the Perdew–Burke–Ernzerhof (PBE) functional. Spin-polarization was included in all the calculations and a damped van der Waals correction was incorporated using Grimme's scheme to better describe the non-bonding interactions. A plane wave cut-off energy of 500 eV was used, and a 3×3×1 Monkhorst-Pack grid k-points was employed. The residual force and energy on each atom during structure relaxation were converged to 0.005 eV $Å^{-1}$ and 10^{-5} eV, respectively. The Hubbard model (DFT+U) introduced by Dudarev et al. was utilized, where the effective U (Ueff) values were set to 4.0 eV for Co, 6.4 eV for Ni and 4.2 eV for Fe, respectively.

The oxygen vacancy formation energy（E_{ov}）is defined as the energy required to remove a neutral oxygen atom from the crystal lattice, creating a stable oxygen vacancy (V_o), E_{ov} values were calculated for four representative models: LC, LSC, LC/NiFe-LDH and LSC/NiFe-LDH.

$$E_{ov} = E_{defect} - E_{perfect} + E_{O2}/2 \quad (S1)$$

Where E_{defect} denotes the total energy of the fully relaxed supercell containing a single oxygen vacancy; E_{defect} denotes the total energy of the fully relaxed pristine supercell with identical lattice vectors and computational parameters; and E_{O2} denotes the corrected total energy of an isolated O_2 molecule in the gas phase.

The ideal work of adhesion W_{ad} is defined as the interface bond energy needed (per unit area) to reversibly separate an interface into two free surfaces:

$$W_{ad} = (E_{perovskite} + E_{NiFe\text{-}LDH} - E_{interface}) / (2A) \quad (S2)$$

Where $E_{perovskite}$ denotes the total energy of the fully relaxed perovskite slab (LC or LSC); $E_{NiFe\text{-}LDH}$ denotes the total energy of the fully relaxed NiFe-LDH slab; $E_{interface}$ denotes the total energy of the corresponding heterointerface system (LC/NiFe-LDH or LSC/NiFe-LDH); and A denotes the interface area.

In the OER, the AEM step and the corresponding Gibbs free energy change can be expressed as follows:

$$* + OH^- \rightarrow *OH + e^- \quad (S3)$$

$$\Delta G_1 = G_{(*OH)} + G_{(H_2)}/2 - G_{(*)} - G_{(H_2O)} \text{ (S4)}$$
$$*OH + OH^- \rightarrow *O + H_2O + e^- \text{ (S5)}$$
$$\Delta G_2 = G_{(*O)} + G_{(H_2)}/2 - G_{(*OH)} \text{ (S6)}$$
$$*O + OH^- \rightarrow *OOH + e^- \text{ (S7)}$$
$$\Delta G_3 = G_{(*OOH)} + G_{(H_2)}/2 - G_{(*O)} - G_{(H_2O)} \text{ (S8)}$$
$$*OOH + OH^- \rightarrow * + O_2 + H_2O + e^- \text{ (S9)}$$
$$\Delta G_4 = G\_{(O_2)} + G_{(*)} + G_{(H_2)}/2 - G_{(*OOH)} \text{ (S10)}$$

The LOM step and the corresponding Gibbs free energy change can be expressed as follows:

$$* + OH^- \rightarrow *OH + e^- \text{ (S11)}$$
$$\Delta G_1 = G_{(*OH)} + G_{(H_2)}/2 - G_{(*)} - G_{(H_2O)} \text{ (S12)}$$
$$*OH + OH^- \rightarrow *O + H_2O + e^- \text{ (S13)}$$
$$\Delta G_2 = G_{(*O)} + G_{(H_2)/2} - G_{(*OH)} \text{ (S14)}$$
$$*O \rightarrow *(V_O)O_2 \text{ (S15)}$$
$$\Delta G_3 = G_{(*(V\_O)O_2)} - G_{(*O)} \text{ (S16)}$$
$$*(V_O)O_2 + OH^- \rightarrow *(V_O)OH + O_2 + e^- \text{ (S17)}$$
$$\Delta G_4 = G_{(*(Vo)OH)} + G_{(O_2)} + G_{(H_2)}/2 - G_{(*(Vo)O_2)} - G_{(H_2O)} \text{ (S18)}$$
$$*(V_O)OH + OH^- \rightarrow * + H_2O + e^- \text{ (S19)}$$
$$\Delta G_5 = G\_{(*)} + G\_{(H_2)}/2 - G_{(*(Vo)OH)} \text{ (S20)}$$

The Gibbs free energy of adsorption is adjusted by the zero-point energy (ΔZPE) and entropy (ΔS):

$$\Delta G = \Delta E + \Delta ZPE + T\Delta S \text{ (S21)}$$

The energy difference between the reactants and products in a chemical reaction is represented as ΔE, which includes the zero-point energy adjustment (ΔZPE). Furthermore, the variation in vibrational entropy at a given temperature T is indicated as ΔS.

To evaluate the performance of OER, the overpotential η_{OER} could be calculated using the following method:

For the AEM pathway (four electron-transfer steps):

$$\eta_{OER} = \max(\Delta G_1, \Delta G_2, \Delta G_3, \Delta G_4,)/e - 1.23 \text{ (S22)}$$

For the LOM pathway (five steps):

$$\eta_{OER} = \max(\Delta G_1, \Delta G_2, \Delta G_3, \Delta G_4, \Delta G_5)/e - 1.23 \text{ (S23)}$$

Here, max(…) refers to the step with the highest Gibbs free energy change, which corresponds to the thermodynamic rate-determining step (RDS) for each pathway.

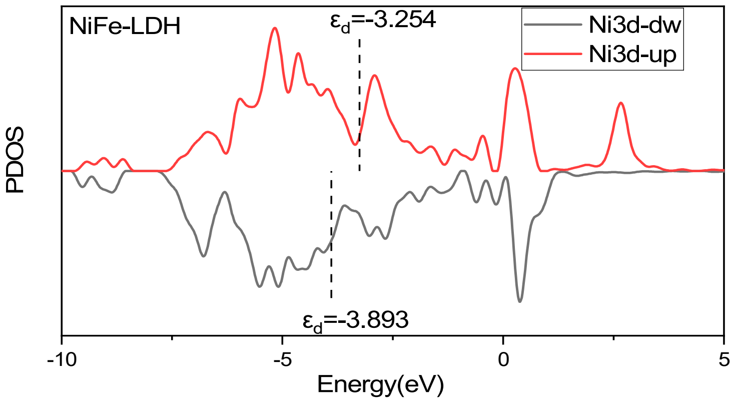

Figure S1. Spin-resolved PDOS of Ni 3d orbitals and the corresponding d-band center (εd) for pristine NiFe-LDH.

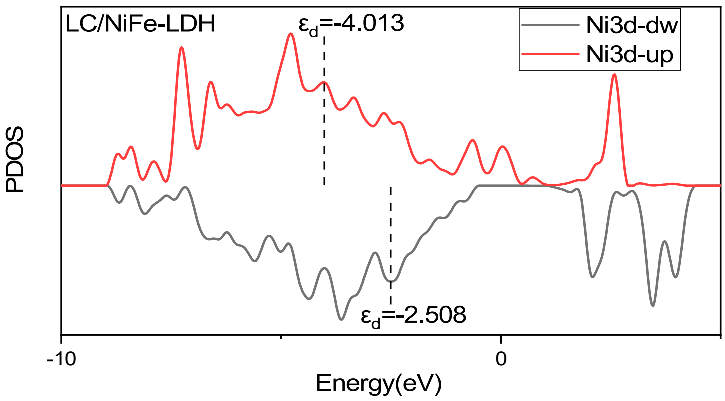

Figure S2. Spin-resolved PDOS of Ni 3d orbitals and the corresponding d-band center (εd) for LC/NiFe-LDH.

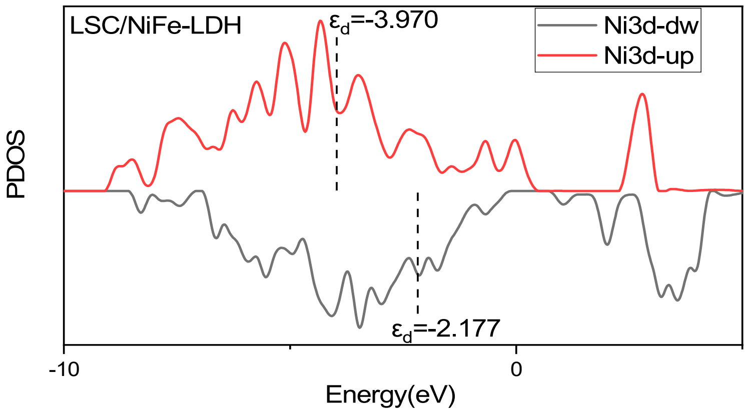

Figure S3. Spin-resolved PDOS of Ni 3d orbitals and the corresponding d-band center (εd) for LSC/NiFe-LDH.

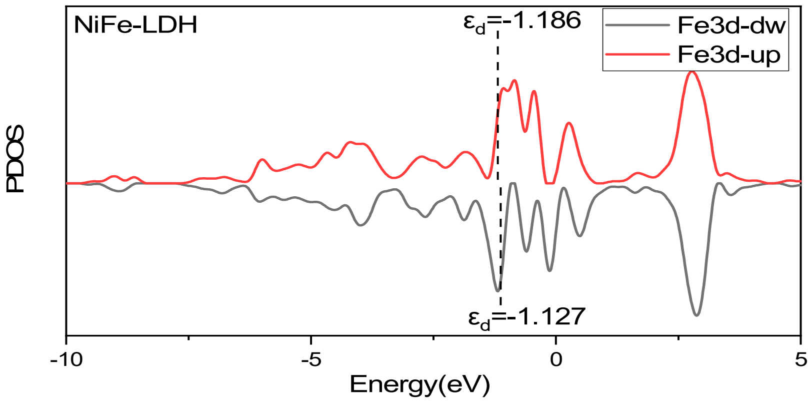

Figure S4. Spin-resolved PDOS of Fe 3d orbitals and the corresponding d-band center (εd) for pristine NiFe-LDH.

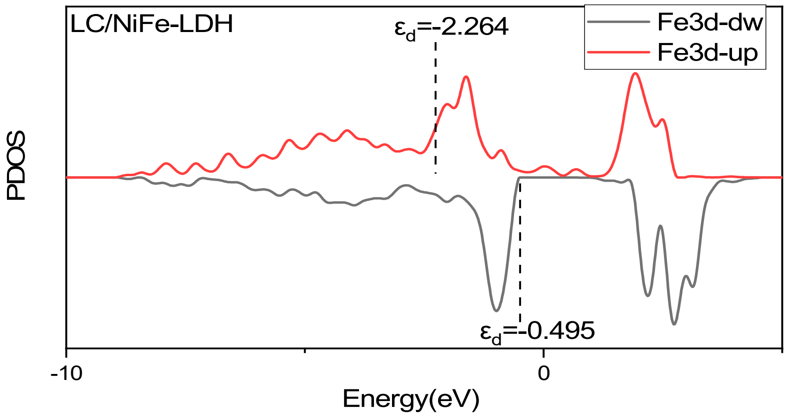

Figure S5. Spin-resolved PDOS of Fe3d orbitals and the corresponding d-band center (εd) for LC/NiFe-LDH.

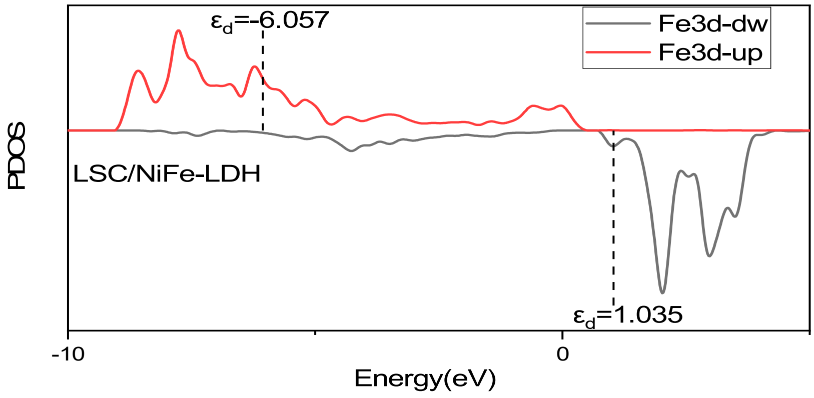

Figure S6. Spin-resolved PDOS of Fe3d orbitals and the corresponding d-band center (εd) for LSC/NiFe-LDH.

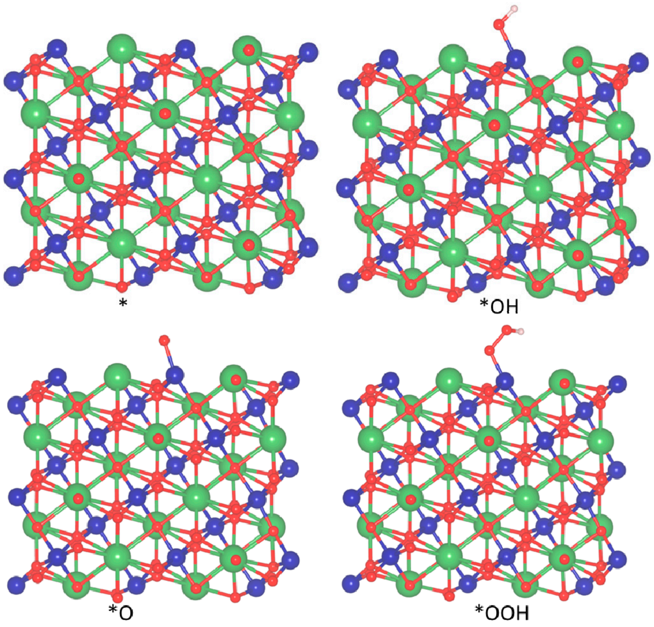

Figure S7. Structures of the key intermediates on the associative electrode mechanism (AEM) pathway of LC.

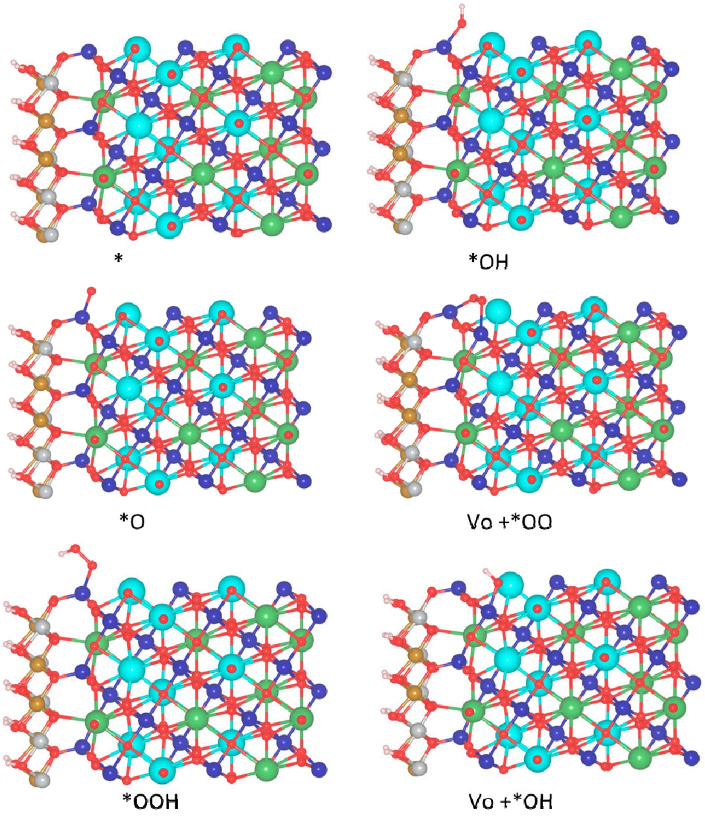

Figure S8. Structures of the key intermediates on the associative electrode mechanism (AEM) and lattice oxygen mechanism (LOM) pathway of LSC/NiFe-LDH.

Table S1. Calculated oxygen vacancy formation energies (E_{ov})

**Catalyst**	**E_{ov}（eV）**
LC	1.89
LSC	1.37
LC / NiFe-LDH	1.73
LSC / NiFe-LDH	1.04

Table S2. Relative abundance of various oxygen species based on O 1s XPS analysis

Perovskite Catalysts		O 1s				Area ratio
		O^{2-}	O_2^{2-}/O^-	OH^-/O_2	H_2O	O_2^{2-}/O^- / $O^{2-}+OH^-/O_2+H_2O$
LC	Position [eV]	528.83	529.89	531.18	532.00	0.051
	Proportion [%]	37.64	4.82	31.63	25.91	
LSC	Position [eV]	528.81	529.6	531.17	532.15	0.149
	Proportion [%]	21.55	12.95	40.39	25.11	
LC/NiFe	Position [eV]	530.99	530.11	531.95	533.31	0.271
-LDH	Proportion [%]	7.33	15.48	39.35	37.84	
LSC/NiFe	Position [eV]	530.11	531.13	531.67	533.38	0.301
-LDH	Proportion [%]	5.19	21.41	42.25	31.17	

Table S3 Comparison of ECSA values for different OER catalysts in alkaline solutions

Catalyst	C（F/g）	ECSA (m^2/g)
LC	6.29	10.48
LSC	7.98	13.29
LC / NiFe-LDH	17.02	28.37
LSC / NiFe-LDH	19.63	30.67

Table S4. OER activities of this work at 10 mA cm^{-2} was compared with that of previously reported similar catalysts in 1 M KOH solution

**Catalyst**	**Overpotential (mV vs. RHE)**	**Tafel slope (mV dec^{-1})**	**Electrode**
**LC / NiFe-LDH**	**237.8**	**90.64**	**Glassy carbon**
**LSC / NiFe-LDH**	**260.1**	**85.06**	**Glassy carbon**
$(La_{0.8}S_{r0.2})_{0.9}Co_{0.1}Fe_{0.8}Ru_{0.1}O_{3-\delta}$	347	54.65	Glassy carbon
$La(CrMnFeCo_2Ni)O_3$	325	51.2	Nickel foam
$(La_{0.6}Sr_{0.4})(Co_{0.2}[FeMnNiMg]_{0.8})O_3$	320	45	Glassy carbon
S-doped $LaCoO_3$	364	126.7	Glassy carbon

$LaCo_{0.94}Pt_{0.06}O_{3-\delta}$	454	86	Glassy carbon
$La_{0.96}Ce_{0.04}CoO_3$	380	80	Glassy carbon
$Pt/LaCoO_3$	427	92	Glassy carbon
$LaCo_{0.8}Ru_{0.2}O_3$	460	43	Glassy carbon
$SrCo_{0.9}Ru_{0.1}O_3$	360	113	Glassy carbon
$La_{1-x}Ce_xNiO_3$	270	45	Carbon paper
0.1Ce–$LaCoO_3$	343	64	Nickel foam
5.6 at. % Ce-doped $LaCoO_3$	390	112	Glassy carbon
Ru-Doped NiFe LDH	246	67.2	Carbon paper
Co_2CrO_4@Cr-NiFe LDH/CF	257	72	Carbon Fiber
Ir doped NiFe-LDH	246	46.7	Carbon cloth
30%Ce–NiFe-LDH	242	34	Glassy carbon

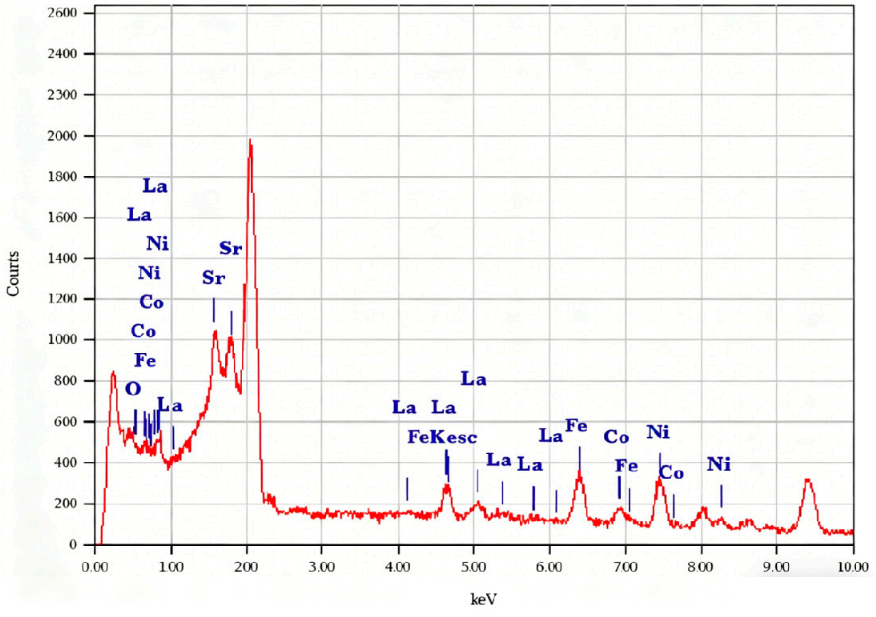

Figure S9. EDS Quantitative Analysis Spectrum of LSC/NiFe-LDH Composite Material

Table S5. EDS quantitative analysis of the LSC/NiFe-LDH composite based on mapping data in Figure S9

Elements	Mass (%)
O	0.7
Fe	21.75
Co	10.39
Ni	39.06
Sr	9.52
La	18.58

Note: The low oxygen content is typical for EDS analysis of oxides due to the limited sensitivity to light elements.

Table S6. Crystallite sizes calculated from the perovskite (110) diffraction peak using the Scherrer equation.

**Sample**	**FWHM (°)**	**Crystallite size (nm)**
LC	0.26	31.53
LSC	0.34	24.11
LC/NiFe-LDH	0.25	32.78
LSC/NiFe-LDH	0.24	34.15

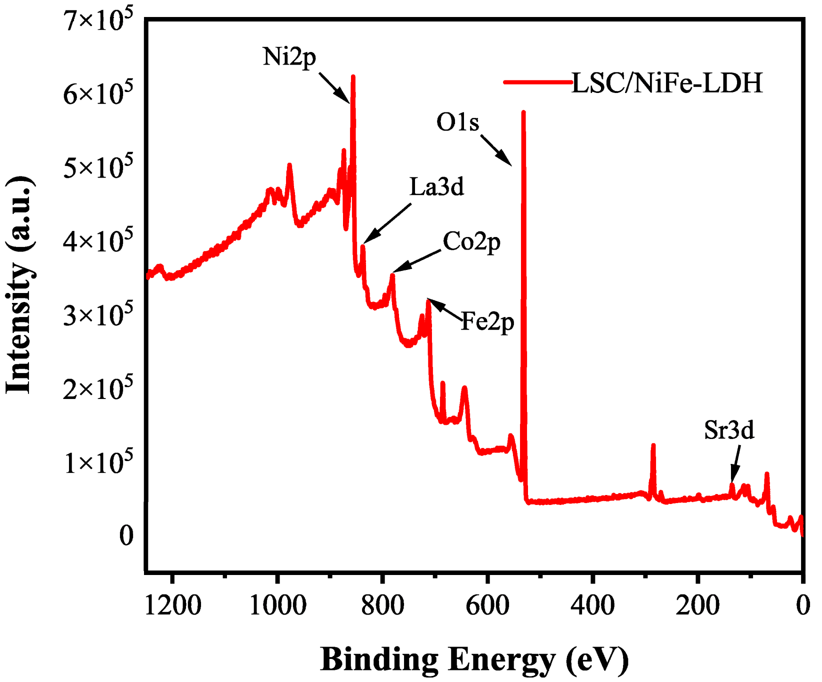

Figure S10. XPS Characterization: Wide-scan XPS survey of the LSC/NiFe-LDH sample.

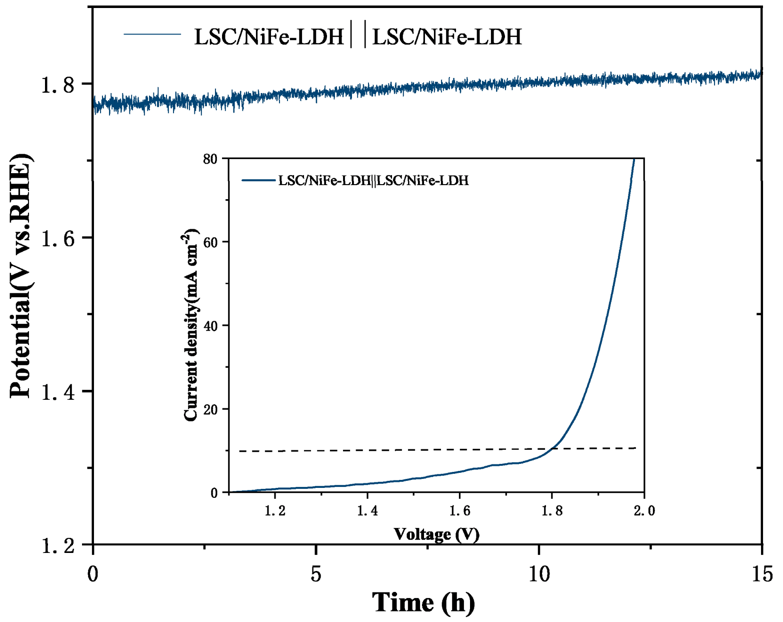

Figure S11. Chronopotentiometry curves of the as-prepared electrocatalysts at a constant current density of 10 mA cm^{-2}; Inset: Overall water splitting polarization curves.

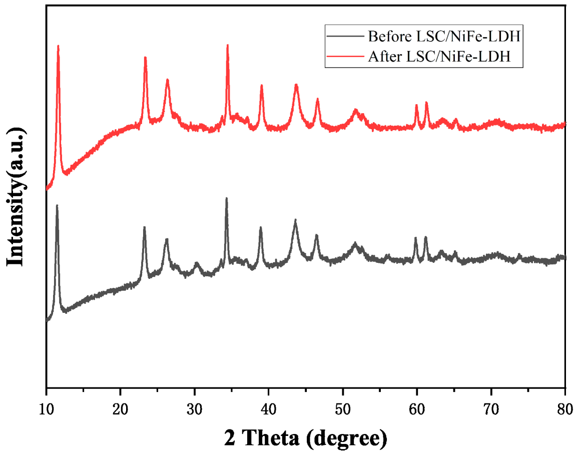

Fig. S12 XRD comparison of LSC/ NiFe-LDH before and after reaction

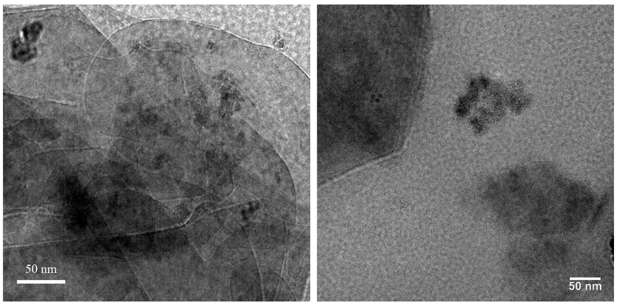

Fig. S13 TEM comparison of LSC/NiFe-LDH before (a) and after (b) reaction
